# Improved anticancer delivery of paclitaxel by albumin surface modification of PLGA nanoparticles

**DOI:** 10.1186/s40199-015-0107-8

**Published:** 2015-04-23

**Authors:** Mehdi Esfandyari-Manesh, Seyed Hossein Mostafavi, Reza Faridi Majidi, Mona Noori Koopaei, Nazanin Shabani Ravari, Mohsen Amini, Behrad Darvishi, Seyed Nasser Ostad, Fatemeh Atyabi, Rassoul Dinarvand

**Affiliations:** Nanotechnology Research Centre, Faculty of Pharmacy, Tehran University of Medical Sciences, Tehran, Iran; Novel Drug Delivery Lab, Department of Pharmaceutics, Faculty of Pharmacy, Tehran University of Medical Sciences, Tehran, Iran; Department of Bioengineering, University of California, Riverside, CA USA; Medical Nanotechnology Department, School of Advanced Technologies in Medicine, Tehran University of Medical Sciences, Tehran, Iran; Department of Medicinal Chemistry, Faculty of Pharmacy, Tehran University of Medical Sciences, Tehran, Iran; Department of Toxicology and Pharmacology, Faculty of Pharmacy, Tehran University of Medical Science, Tehran, Iran

**Keywords:** PLGA, Surface modified nanoparticles, Drug delivery, Albumin, Paclitaxel

## Abstract

**Background:**

Nanoparticles (NPs) play an important role in anticancer delivery systems. Surface modified NPs with hydrophilic polymers such as human serum albumin (HSA) have long half-life in the blood circulation system.

**Methods:**

The method of modified nanoprecipitation was utilized for encapsulation of paclitaxel (PTX) in poly (lactic-co-glycolic acid) (PLGA). Para-maleimide benzoic hydrazide was conjugated to PLGA for the surface modifications of PLGA NPs, and then HSA was attached on the surface of prepared NPs by maleimide attachment to thiol groups (cysteines) of albumin. The application of HSA provides for the longer blood circulation of stealth NPs due to their escape from reticuloendothelial system (RES). Then the physicochemical properties of NPs like surface morphology, size, zeta potential, and in-vitro drug release were analyzed.

**Results:**

The particle size of NPs ranged from 170 to 190 nm and increased about 20–30 nm after HSA conjugation. The zeta potential was about -6 mV and it decreased further after HSA conjugation. The HSA conjugation in prepared NPs was proved by Fourier transform infrared (FT-IR) spectroscopy, faster degradation of HSA in Differential scanning calorimetry (DSC) characterization, and other evidences such as the increasing in size and the decreasing in zeta potential. The PTX released in a biphasic mode for all colloidal suspensions. A sustained release profile for approximately 33 days was detected after a burst effect of the loaded drug. The in vitro cytotoxicity evaluation also indicated that the HSA NPs are more cytotoxic than plain NPs.

**Conclusions:**

HSA decoration of PLGA NPs may be a suitable method for longer blood circulation of NPs.

## Background

Different scientists including pharmaceutics, chemists, biologist, and nanotechnologist have been working indefatigably to defeat cancer. A major interest in this area is to improve drug targeting towards tumor cells and decrease the unwilling effects of chemotherapeutics [1-3]. Nanotechnology is very promising in this field and increases the efficacy of targeting by introducing passive and active targeting [4,5].

The interest on utilizing NPs formulated from biodegradable and biocompatible polymers such as the most commonly used PLGA are rising rapidly [6]. These NPs are broadly studied as anticancer delivery systems since it has special characteristics such as controlled release and biocompatibility [6].

A new approach to evade the short half-life of the conventional drug and allow targeted delivery to tumor cells is drug targeting achieved by size engineering and surface modification [7,8]. Vasculatures in tumor presents several irregularities in contrast with normal vessels resulting in enhanced permeation and retention (EPR) effect [9,10] and this will cause the nanoparticles with diameters less than 100 nm being selectively taken up by tumor Vasculatures [8,11]. However, the drug bio-distribution profile of the cytotoxic drugs change massively while they are incorporated with NPs, because the modified particles are swiftly opsonised and massively cleared by mono nuclear phagocytes system (MPS) [10-12]. Surface modification of particles with hydrophilic polymers like polyethylene glycol (PEG) and albumin leading to the development of long-circulating and stealth particles for delivery of anticancer drugs [13,14]. Furthermore, the lack of lymph vessels and higher interstitial fluid pressure in the most tumors than normal ones causes inefficient removal of interstitial fluid and soluble macromolecules [15]. Therefore, the NPs mount up in the interstitium which retards their uptake (EPR effect), unless those particles are degraded [16,17].

The HSA coated NPs were prepared in two ways. First, non-covalent interactions where HSA molecules only saturate the surface without any covalent linkage [11] and second, albumin conjugated particles were synthesised via reaction between ξ-amino groups of lysine residues and the protein ligand with aldehyde functional or carboxylic acid [18,19]. The second method is more common.

Accordingly, we have developed a novel strategy that benefit from high efficiency and selectivity of the thiol. In this study we did a site-specific conjugation on the HSA that in spite of the fact that it minimize a loss in biological activity of it but meanwhile decrease immunogenicity. It happens because reagents that specifically react with the thiol group of cysteines, and the number of free cysteines on the surface of a protein is much less [15]. HSA conjugation to surface of NPs was done through the disulphide bonds between the HSA and the paramaleimido benzoic hydrazid (PMBH) derivative of PLGA. The encapsulation efficiency (EE), drug release, and morphology of nanoparticles were then investigated. At last cyto-toxicity of PTX loaded NPs was studied using 3-(4,5-dimethyathiazol-2-yl)-2,5-diphenyltetrazoliumbromide (MTT) assay.

## Materials and methods

### Materials

PLGA (50:50, M_W_: 48000 g/mol) with carboxyl end group and HSA were purchased from Sigma company. N, N’-dicyclohexylcarbodiimide (DCC), N–hydroxysuccinimide (NHS), and 3-(4,5-dimethyathiazol-2-yl)-2,5-diphenyltetrazoliumbromide (MTT) were purchased from Sigma-Aldrich (St. Louis, MO, USA). PMBH, Na_3_PO_4_, NaH_2_PO_4_, NaOH, sodium bicarbonate and also NaCl was obtained from Merck. PTX purchased from Cipla Company. Dulbecco’s modified eagle’s medium (DMEM), penicillin, streptomycin antibiotic mixture and fetal bovine serum (FBS) were obtained from Life technologies (grand Island, NY, USA). Polyvinyl alcohol (PVA) was acquired from Acros (Geel, Belgium). 2-(N-morpholino ethane sulfonic acid) (MES) was purchased from Fluka (St. Louis, MO, USA). All other solvents and reagents which are not stated were from Merck (Darmstadt, Germany).

## Methods

### Synthesis of PLGA with functional group of maleimide

Maleimide-functionalized copolymer PLGA was synthesized using the conjugation between paramaleimido benzoic hydrazid (PMBH) and PLGA–COOH. PLGA–COOH (5 g, 0.1 mmol) in 10 ml of methylene chloride was changed to PLGA–NHS with surfeit of N-hydroxysuccinimide (NHS, 135 mg, 1.1 mmol) in the presence of N, N’-dicyclohexylcarbodiimide (230 mg, 1.1 mmol). Then, 0.42 mol PMBH was added to the solution of activated PLGA and the reaction was allowed to proceed overnight on magnetic stirrer. The mixture was evaporated using rotary evaporator and the prepared film of PLGA-PMBH polymer was washed properly using de-ionized water and dried naturally for about two weeks. The synthesized polymer was assessed using H-NMR and FT-IR spectroscopy.

### Preparation of PTX-loaded NPs

The method of modified nanoprecipitation was utilized for the preparation of drug encapsulated into particles of PLGA-PMBH [20-22]. In brief, 20 mg of polymer and 1.4 mg of PTX were dissolved in 4 ml of acetone and then injected (rate = 0.5 ml/min) into 16 ml of aqueous phase containing 0.5% PVA as surfactant and emulsified by probe sonication (Misonix, USA) for 5 min with amplitude of 10. Subsequently, the organic solution was evaporated gently on magnetic stirrer (600 rpm) for 9 hours. The NPs were washed and recovered using centrifuge process 25,000 rpm for 30 min (Sigma 3K30, Germany) and then lyophilized at – 40°C for 48 h (Christ Alpha 1–4; Germany). It should be mentioned that during the procedure, Several parameters in NPs preparation such as surfactant concentration, ratio of organic to aqueous, ratio of drug to polymer, and applied external energy witch have critical effects on the eventual size of NPs and drug loading were assessed in this experiment to obtain optimize situation.

### HSA conjugation on the surface of PLGA NPs

5 mg of NPs was dispersed in 4 ml of degassed deionized water using bubbling nitrogen. HSA (10 mg/ml) were dissolved in 5 ml of degassed deionized water which have NaCl 0.15 M (pH 6.2–6.5) instantly before injecting it into the suspension. 1 ml of degassed solution contained ethylene diamine tetra acetic acid (EDTA) 4 mM and NaCl 0.3 M (pH 6.2–6.5) then were added to the suspension under the nitrogen pressure. The mixture was put a side overnight for the conjugation to perform on the stirrer. The HSA conjugated PLGA NPs was purified and the unreacted HSA was removed using centrifuge (18000 rpm, 30 min, 3 times).

### Measurement of size and zeta potential of NPs

Nearly 1 mg of NPs was suspended in 2 ml deionized water using bath sonicator. Mean size and polydispersity index (PDI) of NPs were evaluated using dynamic light scattering (DLS) instrument (Nano ZS, Malvern Instruments, UK). Afterward, samples were placed in an electrophoretic cell and zeta potential was determined.

### Surface morphology

Scanning electron microscopy (SEM, Philips XL 30, Philips, The Netherlands) was used to determine the shape and surface morphology of the produced NPs. NPs were coated with gold under vacuum before scanning electron microscopy.

### FT-IR analysis

To examine the conjugation was done correctly IR analysis. To perform this procedure we prepared a uniform mixture of lyophilized PLGA and PLGA-HSA NPs (separately) and KBr.

### Differential scanning calorimetry (DSC)

Different ratio of physical mixture of raw materials included PLGA, HSA, PTX and also PLGA NPs and PLGA-HSA NPs were weighted equivalently (7 mg) and then sealed in standard aluminum pans. The experiment carried out using (Mettler Toledo, GmbH, Switzerland) in ascending mode (10°C min/min) started from 40°C to 600°C.

### Drug loading and encapsulation efficiency

To determine the drug loading and encapsulation efficiency, PTX entrapped in the NPs was measured by HPLC (Agilent LC1100, Agilent, Tokyo, Japan) at room temperature. The column was C18 column (25 cm × 0.46 cm internal diameter, pore size 5 μm; Teknokroma, Barcelona, Spain). The mobile phase consisted of acetonitrile/water (1/1 v/v). Lyophilized NPs (2.5 mg) were dissolved in acetonitrile (1 ml) (a common solvent for PLGA and drug) and shaken lightly followed by sonication for 6 min. Then, 2 ml of methanol was added to precipitate the polymer. The sample was filtered and drug quantity in filterant was determined by HPLC analysis.

$$ \begin{array}{l} Drug\; Loading\kern0.5em \%=\left(\frac{weight\kern0.5em  of\kern0.5em  drug\kern0.5em  in\kern0.5em NPs}{weight\kern0.5em  of\kern0.5em NPs}\right)\times 100\\ {} Encapsulation\kern0.5em  Efficiency\kern0.5em \%=\left(\frac{weight\kern0.5em  of\kern0.5em  drug\kern0.5em  in\kern0.5em NPs}{weight\kern0.5em  of\kern0.5em  feed\kern0.5em NPs}\right)\times 100\\ {}\end{array} $$

### In vitro drug release

In order to evaluate in vitro release profile of PTX from PLGA and PLGA-HSA NPs, 2.5 mg of lyophilized samples were dispersed in 5 ml phosphate buffer saline solution (PBS, 0.01 M) containing 5% w/v of sodium dodecyl sulphate (SDS) with different pH (5 and 7.4) [21]. Afterward, suspensions poured into dialysis bags (cut off molecular weight 12000 g/mol) and immersed into the 50 ml of PBS with similar pH to the PBS in the bags. Subsequently, beakers placed on a shaker pre-set its temperature on 37°C and 100 cycles per minute for during 33 days because of slow degradation proses of PLGA. For further assessments, all 50 ml of media (PBS) replaced with a same amount of new PBS at predetermined time intervals. The amount of released PTX was determined by HPLC in wavelength of 228 nm.

### In vitro cell viability

MTT test was used to study the in vitro cytotoxicity of the subsequent PTX formulations on cell line of T47D: PTX loaded PLGA-HSA NPs, PTX loaded PLGA NPs, free PTX, and unloaded NPs.

T47D cells were seeded at the density of 1 × 10^4^ viable cells/well in 96-well plates (Costar, Chicago, IL) and it is also incubated for 24 hours to providing enough time for cell attachments. Then the formulation (100 μL, 1–200 nM, and 48 h) was used to substitute the medium. A stock solution made in dimethyl sulfoxide (1 mg/ml PTX) for PTX. The concentration of dimethyl sulfoxide kept under 0.5% since at this concentration it has no effect on proliferation of cells and RPMI-1640 culture medium was used as diluents for preparing the working solution of free PTX drug and NPs. 20 μl MTT (5 mg/ml in phosphate-buffered saline) was added at specified periods of time to each well, and after 3 – 4 hours the culture medium containing MTT solution was eliminated. Then, micro plate reader (570 nm) used to read it after dissolve of formazan crystals in dimethyl sulfoxide (100 μL). At last following equation used to evaluate cell viability:

$$ \mathrm{Cell}\kern0.5em \mathrm{viability}\kern0.5em \left(\%\right)=\left(\mathrm{Ints}/\mathrm{Intcontrol}\right)\times 100 $$

In this equation Ints equal to the colorimetric intensity of cells which is incubated with the samples, and Intcontrol is the colorimetric intensity of cells that incubated with the phosphate-buffered saline only as positive control.

## Results and discussion

### Synthesis of polymer

PLGA functionalized with maleimide group was synthesized and characterized. 1H-NMR and FT-IR analysis was used for confirmation of the primary chemical structure of PMBH–PLGA.

There was overlapping doublets at 1.6 ppm which are a confirmation for the methyl groups of the lactic acid. The multiples peaks at 4.8 ppm and 5.2 ppm correspond to the –CH_2_ of glycolic acid and -CH of lactic acid, respectively. The high complexity of the peaks at 4.8 ppm and 5.2 ppm resulting from different sequences of glycolic acid and lactic in the backbone of polymer. There are also some detectable proton signals from maleimide and phenyl groups. Peaks which were present the hydrogens of linker are very weak compered to peaks present PLGA hydrogens because of the small ratio of linker to PLGA. A triplet peak on 7.2- 7.4 can be interoperating as benzoic hydrogens and a small peak found on 6.6 ppm shows the maleimide’s hydrogens [22].

**Figure 1:**
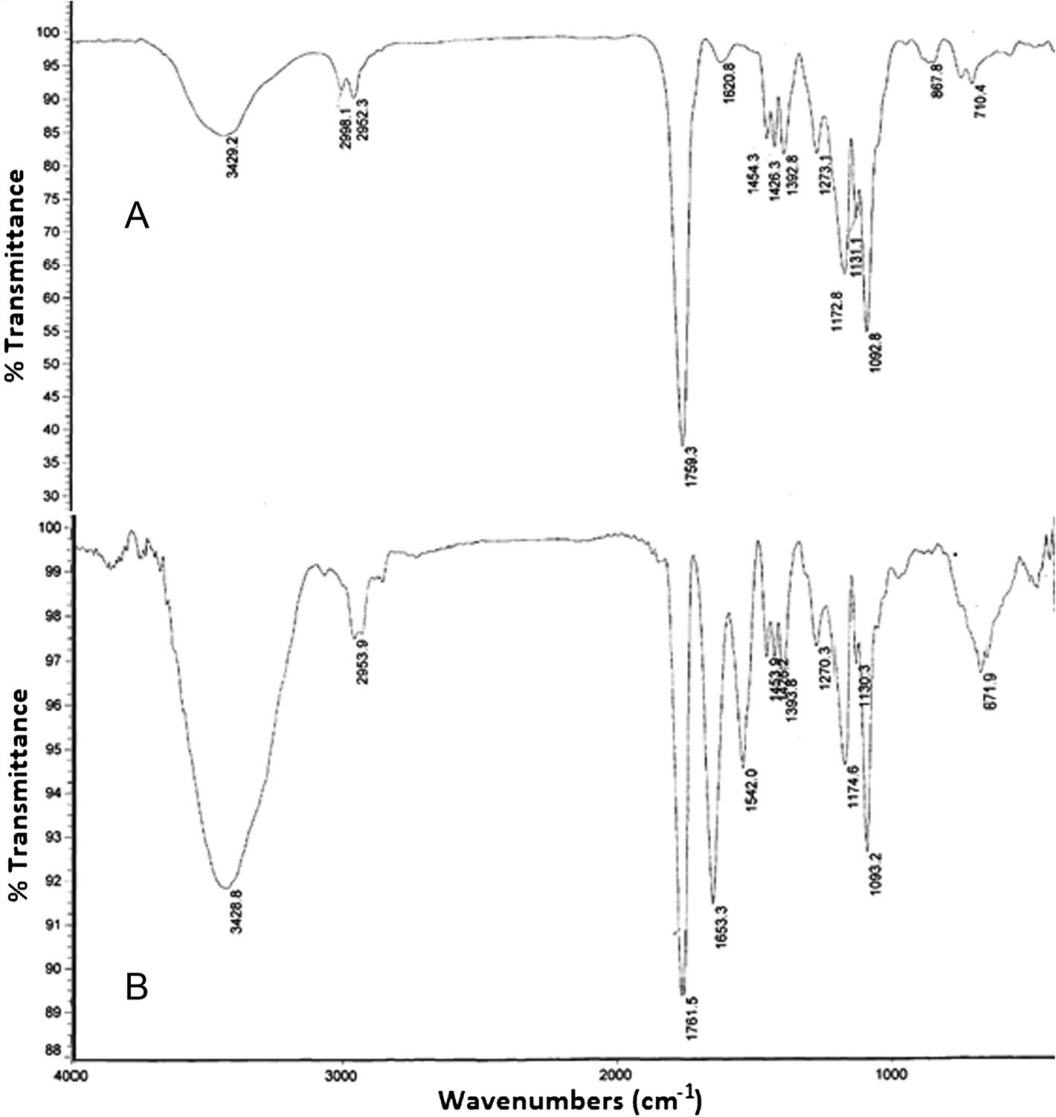
FT-IR spectrum. **(A)** PLGA NPs **(B)** PLGA-HSA NPs.

Conjugation of PLGA-PMBH was shown by FT-IR assessment (Figure 1). Formation of amide bonds are one of the most important reactions in synthesis of PLGA-PMBH. FTIR spectrum of synthesised polymer verified the amide group formation by some peaks, more specifically; the weak bands at 1620 cm^-1^ were assigned to amide bonds. These results verified the formation of PLGA-PMBH was done successfully.

### Nanoparticles characterization

**Figure 2:**
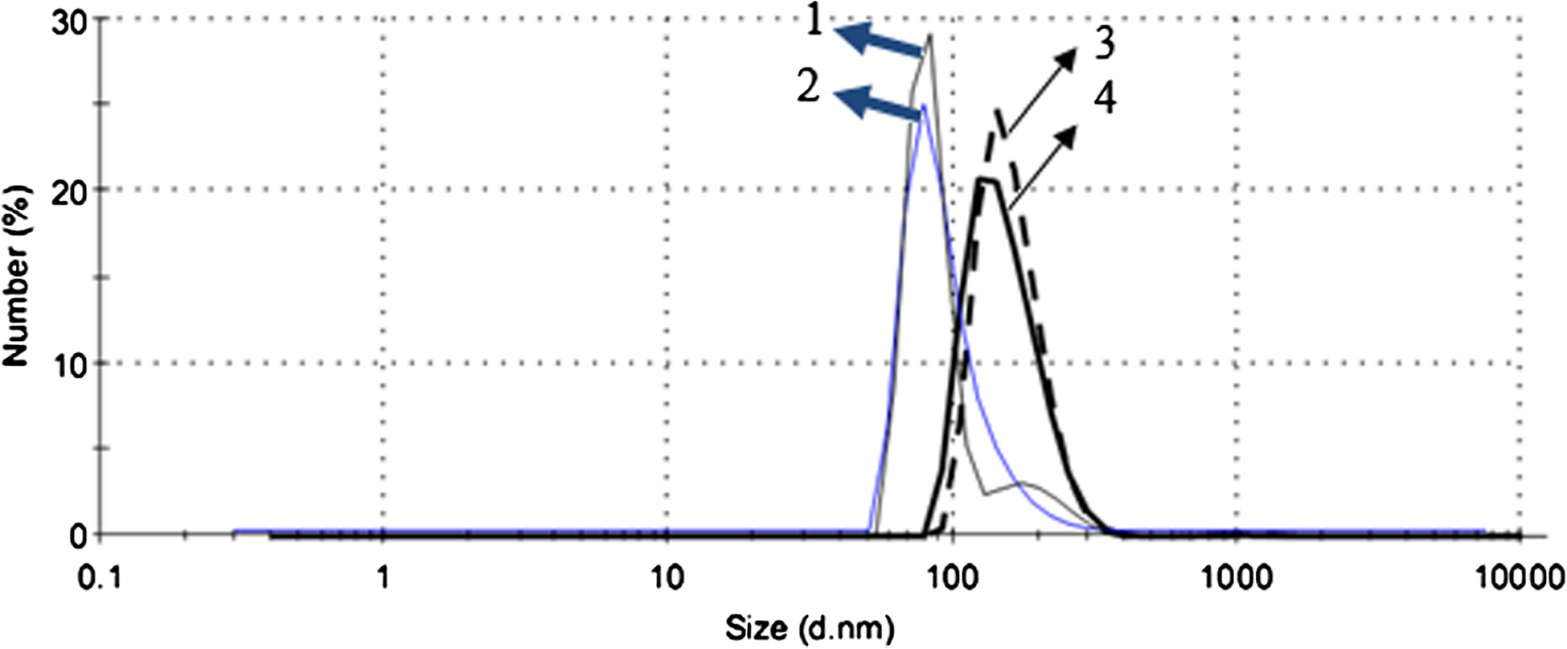
Nanoparticle size increase after HSA conjugation. 1 and 2 are PLGA NPs before HSA conjugation, and 3 and 4 are PLGA-HSA NPs after HSA conjugation.

**Figure 3:**
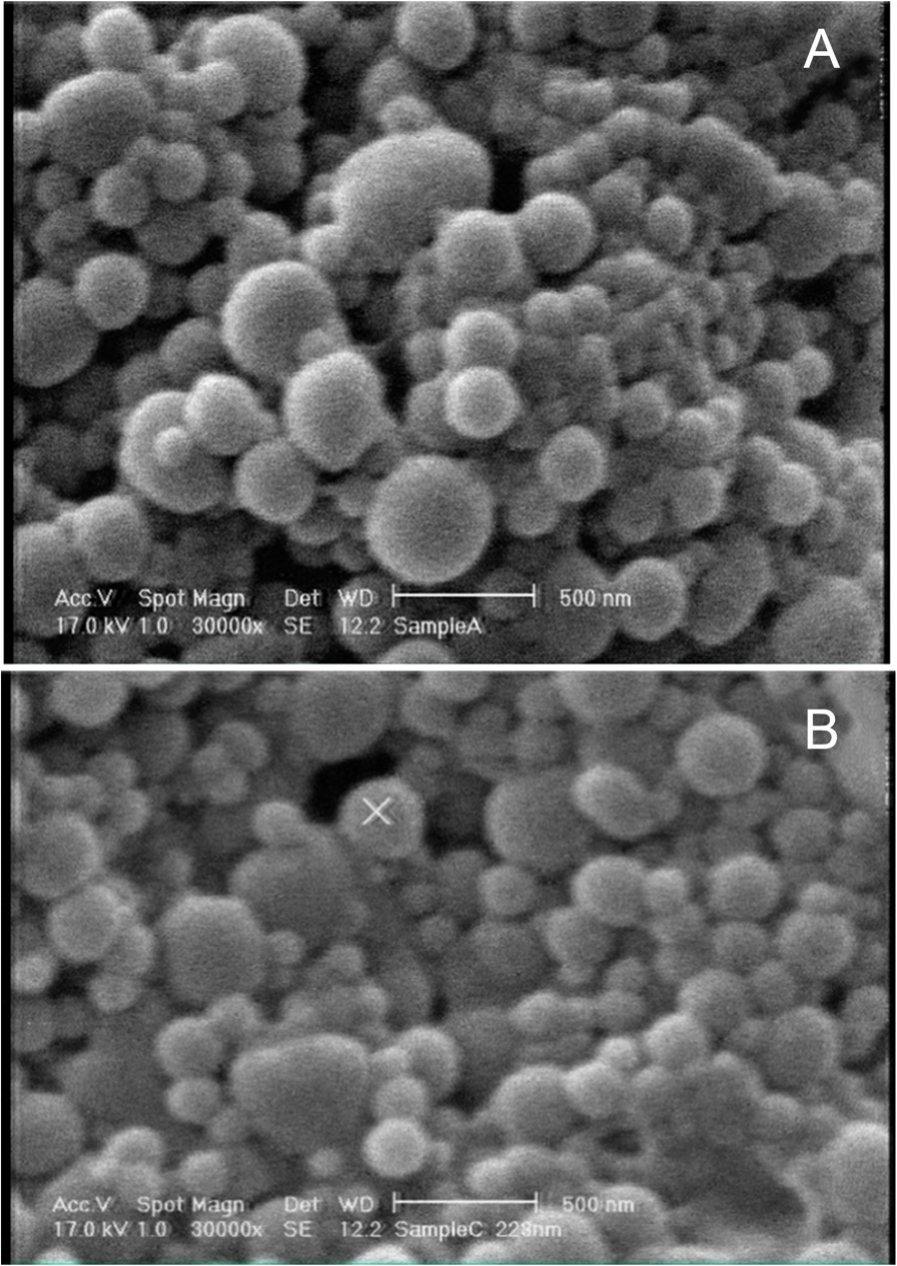
SEM micrographs of NPs with 30,000 times magnification. **(A)** PLGA NPs **(B)** PLGA-HSA NPs.

In the current study the modified nanoprecipitation method was chosen for NPs preparation. Several parameters in NPs preparation such as surfactant concentration, ratio of organic to aqueous, ratio of drug to polymer, and applied external energy have crucial effects on the eventual size of NPs and drug loading, so all of these parameters effects were assessed and the optimized formulations were used to prepare NPs to obtain optimized size [23]. Zeta potential, drug loading, and size of NPs were assessed using DLS and HPLC, respectively (Table 1). The evaluation of NPs size by DLS instrument revealed that the mean particle size of NPs was 190 ± 10 nm and when it was conjugated with HSA it increased about 20–30 nm and reached the mean size of 210 ± 10 nm. Theoretically if HSA with axial ratio of 2.66 nm and hydrodynamic radius of 3.7 nm conjugates in high amount around the surface of NPs, it should increase the size of each NPs roughly 19.7 nm and DLS assessment shows the predicted growth in dimension of each NPs (Figure 2). This phenomenon is clearly observed in SEM pictures that are shown in Figure 3. SEM pictures evaluation shows that NPs have spherical shape and mostly have monodispersed size distribution. The nanoparticle’s zeta potential assessed by DLS display that PLGA NPs have negative charge (-6 mV) and the zeta potential reaches to -13 mV after HSA conjugation in PLGA-HSA NPs. HSA is also is a negative protein and conjugation will reduce the NPs charge [24].

**Table 1:** **Particle size, zeta potential, encapsulation, and loading of NPs before and after conjugation**

**NPs**	**Size (nm)**	**Zeta (mV)**	**Encapsulation %**	**Loading**
PLGA	187.0 ± 10.0	-6.7 ± 1.5	80.1 ± 11.0	10.7 ± 2.6
PLGA-HSA	207.0 ± 5.2	-13.6 ± 1.4	75.4 ± 12.0	8.2 ± 1.3

**Figure 4:**
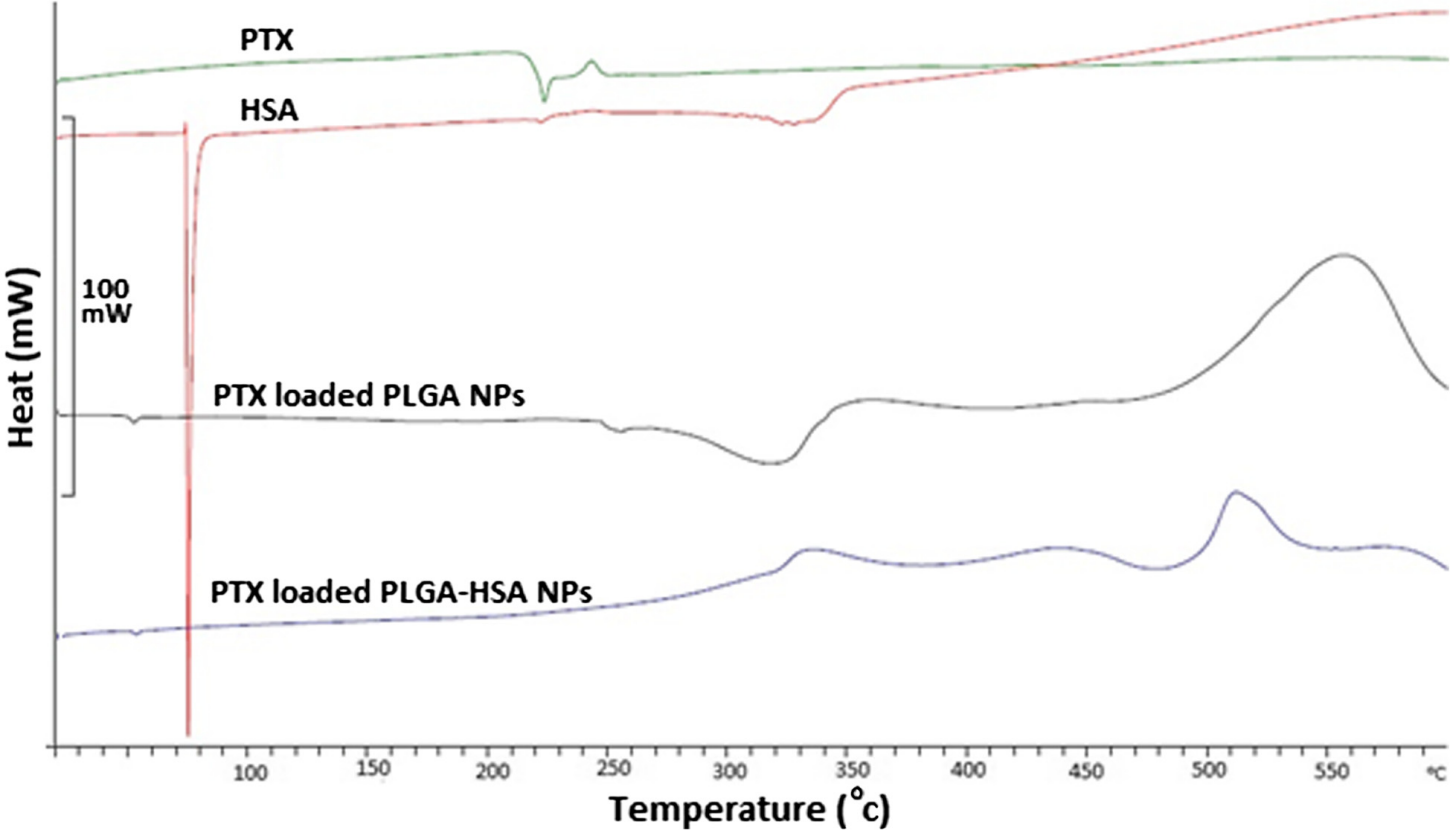
DSC thermograms of PTX, HSA, PLGA NPs, and PLGA-HSA NPs.

DSC thermograms of pure PTX, pure HSA, and PTX loaded PLGA NPs and PTX loaded PLGA-HSA NPs demonstrated in Figure 4. In the drug diagram an endothermic peak observed around 220°C and the absence of that in NPs calorimetric curves proposes the lack of crystallinity after NPs preparation; this suggests that during NPs formation polymer hinders crystallization of PTX and the drug exist in the amorphous state. Other verifications, the differences between PLGA NPs and PLGA-HSA NPs peaks show the conjugation of HSA because of the faster degradation of HSA in PLGA-HSA NPs compared to PLGA NPs [25].

### HSA conjugation

The infrared spectra of PLGA NPs and PLGA-HSA NPs were recorded by using the KBr pellet method (Figure 1). A very sharp peak at 1650 cm^-1^ in PLGA-HSA NPs that obviously point towards amide bonds existed in amino acids in HSA proved the conjugation take place correctly. FT-IR spectrum, faster degradation of HSA in DSC characterization, increasing the size of NPs, and decreasing the zeta potential are reasons which were proved the conjugation of HSA to PLGA-PMBH.

### Drug release profile

**Figure 5:**
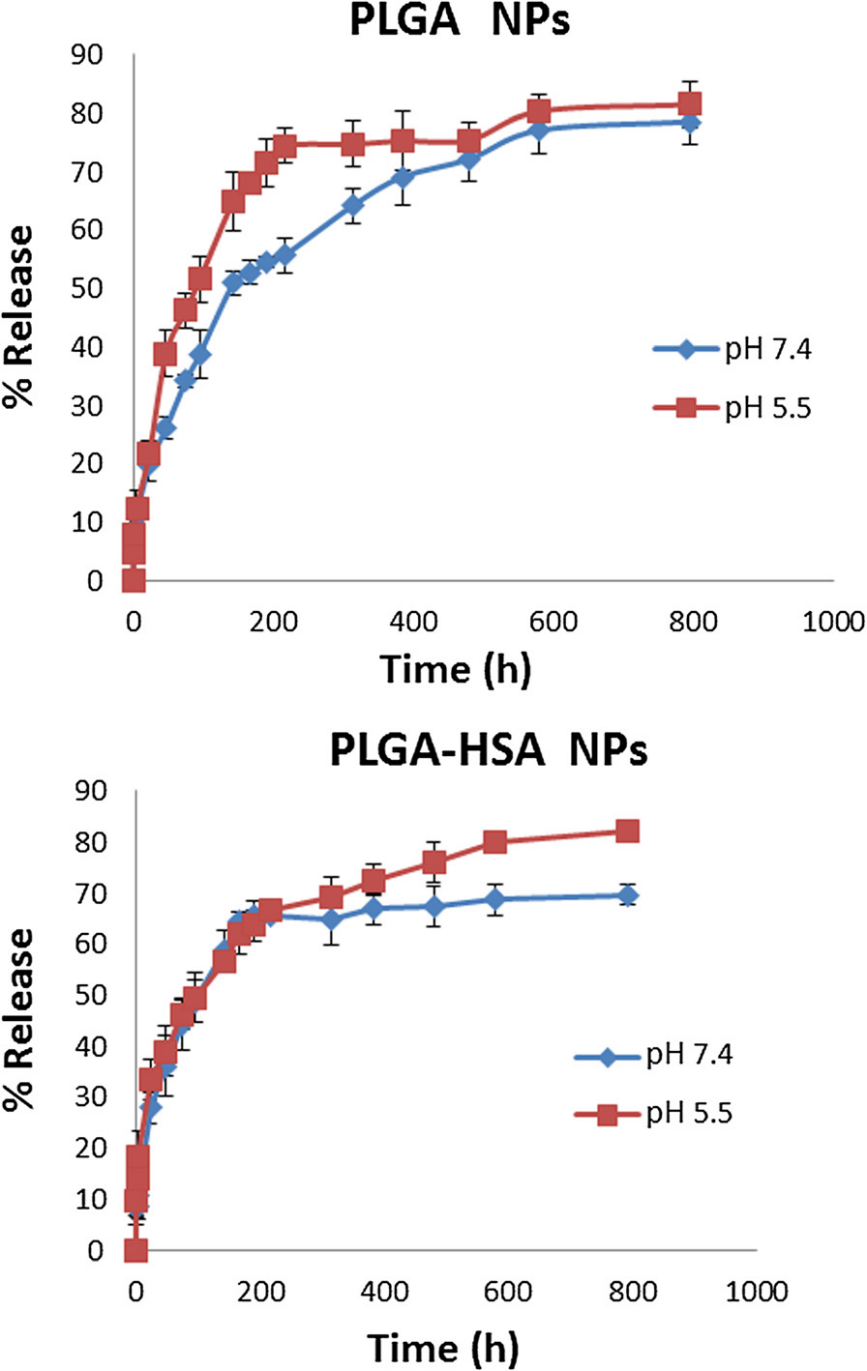
In vitro PTX release profile from PLGA NPs and PLGA-HSA NPs. Data points represent mean ± SD (n = 3).

In vitro drug release was evaluated in PBS with 2 different pH including 5.5 and 7.4 to assess how the different pH may affect the release profile. Acidic pH was chosen to simulate drug release behavior in the cancer cells. It also was examined before and after conjugation of HSA. In all NPs, 80% of loaded PTX released continuously in a sustained manner during 33 days when assessed in pH of 5.5 and about 70% drug released when experiment was carried out in neutral medium. This phenomena shows that drug disperse uniformly inside particles and it comes out of it by diffusion. Figure 5 shows that the drug release in acidic environment is faster than neutral ones for all NPs. Hence, this carrier can release drug faster in acidic surroundings of tumors. Acidic pH enhances hydrolization of ester linkage in PLGA and help encapsulated drug to release in control and sustain manner [24-27].

### In vitro cytotoxicity

**Figure 6:**
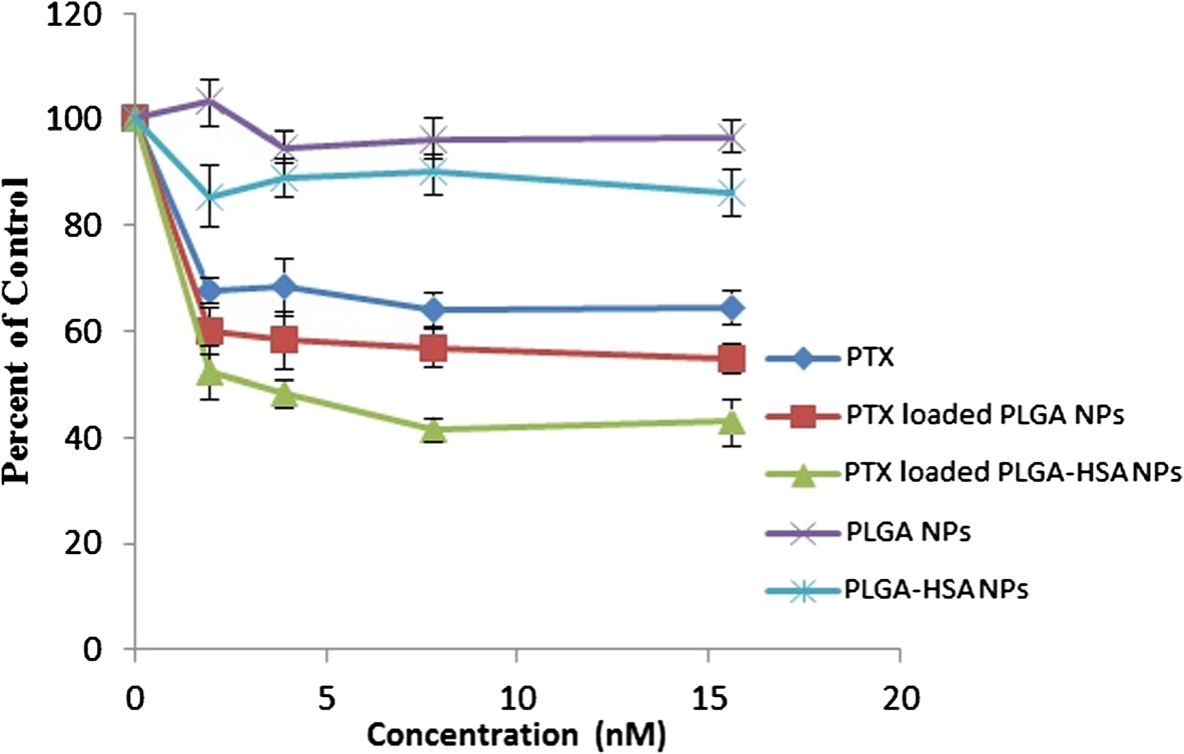
The in vitro cytotoxicity of free PTX, PTX loaded PLGA NPs, and PTX loaded PLGA-HSA NPs with different amount of PTX on T47D breast cancer cells. Data points represent mean ± SD (n = 3).

Figure 6 shows the in vitro cytotoxicity of free PTX, PTX loaded PLGA NPs, and PTX loaded PLGA-HSA NPs with different amount of PTX on breast cancer cells (T47D). Figure 6 illustrate that the cytotoxicity of PTX loaded PLGA-HSA NPs was significantly higher than the free PTX and PTX loaded PLGA NPs. Moreover, PTX loaded PLGA NPs have significantly more cytotoxic effect than free PTX. The percent viability of free PTX, PTX loaded PLGA NPs, and PTX loaded PLGA-HSA NPs were 64%, 54%, and 43% in 15 nM concentration, respectively. The enhancement of antitumor activity of PLGA-HSA NPs may be caused by gp60 (albondin) receptor and caveolar transport which both help these particles to increased transendothelial cell transportation of HSA [23,24]. First, HSA molecules bind to gp60 receptors and this binding activates caveolin. After caveolin configuration, HSA and other plasma constituents transfer transversely the endothelial cell to the interstitial space. Improved intratumor delivery of PTX may also other reason for the increased antitumor activity of PLGA-HSA NPs. Activated gp60 receptors which are specific for HSA help transportation of this molecule into tumor tissues by bypassing blood vessel wall barriers [25]. Unloaded NPs tested to evaluate the effect of polymerization and conjugation on cell viability and statistical analysis proved that these parameters do not affect cell viability.

## Conclusions

Preparation of the PTX loaded PLGA NPs were done by modified nanoprecipitation method. The hydrophobic PLGA NPs were decorated by hydrophilic HSA as novel anticancer delivery system. The PMBH was used as linker for the conjugation of HSA on the surface of PLGA NPs. The drug loading and encapsulation efficiency were 13% and 80%, respectively. Our results demonstrated that by using PMBH as linker and this method of nanoprecipitation, HSA conjugated NPs would be obtained with desired size, morphological, and drug loading properties. The in vitro cytotoxicity also showed that the HSA decorated NPs are more cytotoxic when compared with plain NPs and free anticancer agent, so these NPs can be used successfully in drug delivery of anticancer agents.
